# Political orientation and adherence to social distancing during the COVID-19 pandemic in Italy

**DOI:** 10.1007/s40888-021-00224-w

**Published:** 2021-03-20

**Authors:** Paolo Nicola Barbieri, Beatrice Bonini

**Affiliations:** 1 Prometeia & Centre for Health Economics, University of Gothenburg, Gothenburg, Sweden; 2 Columbia University, New York, USA

**Keywords:** COVID-19, Coronavirus, Political belief, Protest vote, Geolocation data, P16, C55, H7

## Abstract

Many governments have implemented social distancing and lockdown measures to curb the spread of the novel coronavirus (COVID-19). Using province-level geolocation data from Italy, we document that political disbelief can limit government policy effectiveness. Residents in provinces leaning towards extreme right-wing parties show lower rates of compliance with social distancing order. We also find that, during the Italian lockdown, provinces with high protest votes virtually disregarded all social distancing orders. On the contrary, in provinces with higher political support for the current political legislation, we found a higher degree of social distancing compliance. These results are robust to controlling for other factors, including time, geography, local COVID-19 cases and deaths, healthcare hospital beds, and other sociodemographic and economic characteristics. Our research shows that bipartisan support and national responsibility are essential to implement and manage social distancing efficiently. From a broader perspective, our findings suggest that partisan politics and discontent with the political class (i.e., protest voting) might significantly affect human health and the economy.

## Introduction

The novel coronavirus pandemic has posed enormous challenges to our society. While scientists and researchers worldwide are working to find treatments for and vaccines against COVID-19, social distancing measures have proven effective and helpful in curbing the virus’s spread (Engle et al. 2020; Fowler et al. 2020; Bilgin 2020; Abouk and Heydari 2020). However, implementing these restrictions can be challenging, especially in democratic regimes (Hall et al. 2020; Studdert and Hall 2020; Gostin and Hodge 2020). Evidence suggests that countries with more democratic political institutions experienced more per-capita deaths than less democratic countries do. One explanation is that democratic political institutions generally have a bureaucratic disadvantage in responding quickly to pandemics (Cepaluni et al. 2020). In Europe, divergent national responses to COVID-19 reflect different national preferences and political legitimacy. The current pandemic has exposed European weaknesses in coordinated responses to outbreaks, primarily because of the continent’s diverse ideological makeup and deeply-rooted democratic nature (Pacces and Weimer 2020; Anderson et al. 2020). At the same time, the effectiveness of self-quarantine and social distancing orders depends primarily on individual behaviors, especially in cases where governmental coercion cannot be entirely enforced (Parmet and Sinha 2020).

Understanding the determinants of the tendency of compliance behavior might then be an essential step (i) to explain why the virus has spread differently across countries; (ii) to predict its future pattern at least before an effective medical treatment and/or a vaccine are found and administered on a mass scale and (iii) to identify what does not work and to make theoretical prediction more realistic.

The inclination to disobey lockdown measures is traceable to several political factors. First, politicians’ messages are inconsistent: some have adopted a more radical approach, while others have downplayed the epidemic (Adolph et al. 2020; Herrera and Ordoñez 2020). The media have also played a fundamental role in conveying specific messages about the health crisis (Bursztyn et al. 2020). Many countries have experienced conflicting messages about the novel coronavirus’s seriousness, especially in the early stages. To make matters worse, many contrasting views about the efficacy of the use of face masks, gloves, and social distance in general, have been presented even by local scientists and medical experts (Greenhalgh et al. 2020; Feng et al. 2020; Javid et al. 2020). Therefore, in many cases, citizens might be left disoriented about the severity of the epidemic (Wang et al. 2020). The pandemic has also been politicised to the point where even scientific information is systematically disregarded.

Other potential factors might also explain the level of compliance to state-orders. For instance, psychological factors linked to personality (Durante et al. 2020; Bish and Michie 2010) can make one more or less likely to obey rules. Other factors include individual risk aversion (Barrios and Hochberg 2020) and trust in politicians (Bargain and Aminjonov 2020) or the scientific community (Brzezinski et al. 2020; Battiston et al. 2021). Just as an individual and national responsibility affect the effectiveness of social distance, civic sense and public spirit can play a role in encouraging desirable habits.

Studying Italians’ willingness to comply with social distancing measures is particularly interesting given that recent works have extensively documented the recent rise of populism and increased success of far-right parties (Colantone and Stanig 2018; Caselli et al. 2020; Barone et al. 2016). At the same time, other works have shown that political stances and partisan bias can help explain the differential levels of adherence to “stay-at-home” orders (Painter and Qiu 2020; Allcott et al. 2020). We combine these two emerging literatures, and provide, to the best of our knowledge, the first empirical evidence of the political determinants of behavioral responses to Italy’s lockdown measures. We aim to test whether local political preferences mediate the effectiveness of government’s containment measures by impacting on the overall reduction in individual local proximity.

Specifically, we consider human mobility to measure the citizens’ compliance, or the lack thereof, with the various containment rules enacted by the government. 1 Exploiting data on individual mobility at the provincial level and considering electoral results of the 2018 national Italian elections, we find that higher vote shares for the extreme right-wing party predict lower adherence to the lockdown measures and a lower extra reduction in social distancing relative to the means. We also examine protest voting and show that political discontent is associated with a counteractive disregard for social distancing rules during the lockdown. Finally, we find that political support for the current legislation, as measured by vote shares for Movimento 5 Stelle (M5S), causes higher extra adherence to lockdown measures. These results are robust to alternative modelling assumptions.

Our findings support the thesis that political (mis-)belief influences compliance with lockdown measures, eventually affecting the effectiveness of the precautionary measures themselves. Our results suggest that provincial differences in political beliefs are significant to consider in designing nationwide policies. Our analysis helps policymakers understand behavioral responses to the pandemic and redesign (where needed) social distancing and lockdown policies for the subsequent waves of the COVID-19 pandemic. Another policy implication of our results is that bipartisan support is needed to support nationwide social distancing interventions. Any attempt to use COVID-19 news for political and ideological purposes might erode trust in political institutions and create a general state of information crisis (Lovari 2020).

The remainder of the paper is organised as follows: Section 2 reviews the literature; Section 3 describes the timeline of the events related to the COVID-19 outbreaks in Italy; Sections 4 and 5 respectively present the data employed and the econometric model used; Section 6 reports the results and Section 7 concludes.

## Literature

Although research about the current COVID-19 crisis and the pandemic response are recent, they are not limited, and there are many strands of the literature related to our work.

First, this analysis is part of a broader literature investigating heterogenous responses to pandemics (Crouse Quinn 2008; Blendon et al. 2008; Vaughan and Tinker 2009; Fineberg 2014). Many works find that demographic characteristics, such as gender, income, and location, influence the adoption of recommended public health behaviors (Bish and Michie 2010; Bults et al. 2011; Chuang et al. 2015). Perception of risks, how the media transmit information, and the general level of trust also plays a crucial role (Ibuka et al. 2010; van der Weerd et al. 2011; Giuliano and Rasul 2020). Barrios and Hochberg (2020) find that US counties with a higher share of Trump voters have lower perceptions of risk, on average, during the COVID-19 pandemic. This suggests that information treatments and “stay-at-home” orders may not be effective in an ideologically diverse population.

Similarly, the earliest citizen responses to COVID-19 were affected by how its news has been politicized and used for ideological interests (Abbas 2020). Examining politically-charged fake news, Long et al. (2020) find that conservative-media dismissals of hurricane Harvey and Irma’s dangers led to lower evacuation rates for conservatives relative to liberals. For Italy, Lovari (2020) finds that the spread of political misinformation led the Italian Ministry of Health to resort to its official Facebook page to rectify the online community’s misinformation. The scientific community plays a pivotal role in helping the citizen navigate through the overabundance of information that spreads during an epidemic, not to add to confusion and misinformation alongside efforts to counter or challenge it (Cole 2020).

An extensive literature on trust has produced contrasting results. Bargain and Aminjonov (2020) examine whether, in Europe, the compliance to containment policies depends on the level of trust in policymakers before the crisis. Their evidence supports the idea that the degree of compliance with containment rules (i.e., drop in human mobility) is more substancial where the regional level of trust in politicians is high. Evidence in Brodeur et al. (2020) shows that compliance post-lockdown is especially large for trust in the press and relatively smaller for trust in science, medicine, or government. Bicchieri et al. (2020) argue instead that, since practicing social distancing, wearing masks, and staying at home are voluntary and conditional on the behavior of others, they are most affected by what others do (empirical expectations) and what others approve (normative expectations), concluding that country-level trust in science, and not in government, becomes a strong predictor of compliance. According to Cairney and Wellstead (2021), citizens need to trust their elected politicians, who need to base their decisions on scientific experts to understand and respond to the problem. Trust in the scientific community is necessary for cooperation, coordination, social order and to reduce the need for coercive state imposition. Politicians alone cannot solve the current crisis, and they need to rely on their scientific community, which citizens need to trust to support the government more efficiently.^2^ On this last aspect, Battiston et al. (2021), Brzezinski et al. (2020) look at the effect of trust in science and experts using digital trace data from several social media, finding that trust in science, relative to trust in institutions, emerges as a consistent predictor of both knowledge and containment outcomes. Using large field online survey experiments in Italy, Spain, Germany, and the Netherlands, Daniele et al. (2020) conclude that individuals feel more secure in trusting competent experts to design management actions. COVID-19 created a rallying effect around scientific experts and made populist policies lose ground, suggesting an increasing demand for competent leadership. Such findings suggest public health interventions and messaging about risks associated with COVID-19 need to not only be more competent and scientific-grounded than ever but also need to consider that local attitudes towards science may be crucial.

Several studies have also analyzed the effect that media campaigns have in affecting people’s health behaviors in multiple contexts (Austin et al. 2006; Altmann and Traxler 2014). In the COVID-19 pandemic, several studies have discussed reminders’ role in affecting people’s ex-ante intentions (Barari et al. 2020; Everett et al. 2020; Utych and Fowler 2020; Jordan et al. 2020). Falco and Zaccagni (2020) use a randomized control trial in Denmark to study whether different treatments affect people’s intentions and actual behaviors. Their results show that people answer to the messages only when they highlight consequences on the recipient and his/her family, not when they stress effects on the country and society. The campaign seems to affect ex-ante intentions while leaving actual behavior unaltered. Briscese et al. (2020) find that intentions to comply with social distancing measures respond to the length of their extension, highlighting the critical role of expectations in changing willingness to abide by the rules and thus shedding light on the cruciality of consistency and coherence in communication on behalf of the government.

At a deeper level, our study is also linked to the strand of the literature in political psychology, which shows the strong association between personality traits (often measured by the Five-Factor Model of Personality) and voting choices and political participation (Schoen and Schumann 2007; Gerber et al. 2010, 2011; Funk et al. 2013). According to Bakker et al. (2016), low levels of Agreebleness (the personality dimension that includes attributes such as trust, altruism, kindness, affection, and other prosocial behaviors) are associated with voting anti-establishment parties. Barrios et al. (2021) show that voluntary social distancing during the early stages of the COVID-19 in the US and Europe was more significant in areas where most of the population poses a higher sense of duty. Even after some US States began to re-open, high civic capital counties maintained a more sustained level of social distancing, while low civic capital counties did not. Recent work by Durante et al. (2020) also finds evidence that civic capital affects the magnitude of the drop in mobility in Italy during the pandemic. Similarly, prosocial individuals are more likely to follow physical distancing guidelines, stay home when sick, and buy face masks in Campos-Mercade et al. (2020). The authors also show that pro-sociality measured two years before the pandemic predicts health behaviors during the COVID-19 emergency, showing that long-term determinants that cannot be easily changed in the short term are the main predictors of policy-relevant behaviors. This part of the literature, which links political science, psychology, sociology, and public policy, can provide interesting insights into how political beliefs drive individual behaviours, potentially impacting our economies. Our work expands to this literature by shedding light on the relationship between anti-establishment voting and prosocial behavior, as measured by the restriction orders’ level of adoption.

We also adds to the growing body of research that uses geolocation data to study behaviors and social interactions (Psyllidis et al. 2018; Bargain and Aminjonov 2020) and to the works that focus on the effects of political polarization on health behaviors (Iyengar et al. 2019; Montoya-Williams and Fuentes-Afflick 2019). The papers closest to ours investigate the effect that partisan support has had on social distancing in the United States during the current COVID-19 crisis. For instance Painter and Qiu (2020) exploit US geolocation data to show that political beliefs can significantly alter the effectiveness of state-level social distancing orders. They define “aligned” counties as those with the same political affiliation as the governor and “misaligned” counties as those with conflicting political identities, finding that misaligned counties have lower responses to state orders relative to aligned counties, with a stronger effect for misaligned Democratic counties. Engle et al. (2020) also support the evidence that US Democratic counties that did not support Republicans during the last presidential elections obey more local restriction orders in the US. Similarly, Allcott et al. (2020) use location data from a large sample of smartphones to show that areas with more Republicans engage in less social distancing, even after controlling for other factors, including state policies, population density, and local COVID cases and deaths.^3^ Not only political orientation affects how individuals react to social distancing rules but also the pandemic itself might affect political orientation and voting intentions based on whether people account the incumbent politicians responsible for potential inefficient handling of COVID-19 (Achen and Bartels 2016; Fearon 1999). Baccini et al. (2021), for example, look at the potential effect that the pandemic might have had on the 2020 Presidential election in the USA. Similarly, Herrera et al. (2020) find that incumbents’ approval rates increase strongly but drop in countries where COVID cases continue to grow, especially where governments did not implement stringent policies to control the number of infections. These last two papers suggest that voters reward governments that placed more weight on health than short-term economic outcomes. Within the Italian context, Caselli et al. (2020), using mobility data, show that the local labour markets’ characteristics played an important role after the traveling bans were lifted. The catching up with pre-COVID-19 patterns has been stronger in those areas where the labour force is relatively less exposed to the risk of contagion and less likely to work from home.

## Italian COVID-19 history and government containment measures

The first two cases of the novel COVID-19 in Italy were reported on a couple of Chinese tourists on January 30 in Rome at the Spallanzani Institute. On the same day, Italy blocked all flights to and from China indefinitely through an order of the Minister of Health.^4^ The Italian government declared a state of emergency on the January 31, appointing the Civil Protection as the supreme health authority to deal with the pandemic. On February 5 the Head of the Civil Protection, set up a technical-scientific committee, later expanded by an order dated April 18.^5^

The first official case of secondary transmission occurred in Codogno, in the province of Lodi (Lombardy) on February 18. After the first cases were reported in Codogno and the village of Vo’ Euganeo (Veneto), Italian COVID-19 cases and deaths continued to increase rapidly, eventually leading to a severe outbreak, especially in the regions of Lombardy, Emilia-Romagna, Veneto, and Piedmont. To contain these outbreaks, the Council of Ministers passed a decree-law on February 23 with measures that prohibited access in and departures from the municipalities where outbreaks were present, the so-called “Red Zones,” suspending all demonstrations and events.

Subsequently, several and subsequently more stringent decrees were implemented,^6^ until the last one passed on March 9, 2020, implementing the lockdown and the foreclosure of non-essential commercial activities.^7^ The lockdown measures imposed citizens to stay in the municipality in which they were found when the decree was officially published. Such containment measures allowed travels and movements only for proven work needs, absolute urgencies, or for health reasons; they also required people to fill in the forms of self-certification, in which the citizen must report his identification data, declare the place of departure and arrival, and specify the reason for moving.^8^ Other new additional measures were issued to contain and manage the epidemiological emergency from COVID-19, being applicable on the entire national territory. The act imposed the closure of non-essential or strategic production activities. Food, pharmacies, necessities shops, and groceries remained open.^9^ The decree suspended the training sessions of athletes, professionals, and non-professionals within sports facilities of all kinds. All the lockdown measures to combat the spread of coronavirus infection were further extended until mid-April and then until May 3.

With a Prime Ministerial Decree of April 26, the government enacted the measures to contain the COVID-19 emergency of the so-called “phase-two.” The decree provisions apply from May 4 and are effective until June 14, except for the provisions for business activities, which re-open only after May 17. Phase-two offocially ends the lockdown. In fact, while adopting the proper social distancing measures and devices (i.e. face-masks), it allows for several activities, which were previously banned, to re-open (e.g. travel back to home-town if stuck in a different region, visit parents living in the same region, open parks, do outdoor physical activity and restaurant delivery).

Lastly, on June 11, a new DCPM was published, in force from June 15, which further eases containment measures the period of “cohabitation” with the virus (or phase-three). Access to indoor and outdoor places intended for recreational activities with the presence of operators is allowed. Shows open to the public at theaters, cinemas, and concert halls reopened, with a maximum of two hundred spectators indoors and a thousand outdoors, with pre-assigned seats at least one meter apart; the bathing establishments, the wellness and spa centers, the cultural and social centers are reopened; the demonstrations are allowed only in static form. Such DPCM leaves the regions free to loosen or restrict these last measures and postpone them, based on the epidemiological situation of their territories.

## Data

The time window for analysis is February 24–June 26, 2020. Appendix Table 2 reports summary statistics for all variables used in the empirical analysis.

Social distancing For social distancing measures, we use daily data on the average degree of a spatial proximity network collected by Pepe et al. (2020), which assemble a large-scale, de-identified location-based dataset from voluntary users. This dataset’s most salient advantage is its high frequency, which allows researchers to identify various timing variations.^10^

Using such location data, Pepe et al. (2020) derive three metrics of mobility and proximity: (i) the daily origin-destination matrices measuring users’ movements between Italian provinces; (ii) the weekly users’ average radius of gyration by province, capturing the extent of individual movements; (iii) the daily average users’ proximity network, capturing the level of social distancing by province. In this study, we employ the daily average users’ proximity network to capture the level of social distancing by province.^12^

Pepe et al. (2020) compute the average contact rate, or the number of unique contacts made by a person on a typical day, as a proxy of the potential encounters each anonymous user could have in one hour. To this aim, they build a proximity network among users based on the locations they visited and the hour of the day when these visits occurred. They collect data on all users’ positions in a given province and then create a disk of 50 meters around each stop of the users. Finally, if two disks of a pair of different users intersect during the same time window, a link is placed between the two users in the resulting network.^13^ The mean hourly network degree is measured as $k=\dfrac{2E}{N}$, where *E* is the number of edges and *N* is the total number of nodes in the network, including those with $k = 0$. The mean daily degree is obtained by averaging all the 24 values of *k* measured in a day in a given province.

It is important to remark that this is not a close-range contact network. Instead, it captures a looser notion of social mixing at the chosen spatial and temporal scales. A link between two nodes shows the possibility that the corresponding individuals have had a close-range encounter during a day. However, because of the non-uniform sampling, we do not compare the raw values of *k* across provinces^14^ Instead, we will analyze the effect of political orientation on the relative variation *k*. between the pandemic period’s various phases.

Election data To measure Italian residents’ political preferences, we use the province-level voting data from the Internal Ministry of the 2018 Italian general election for seats in the Chamber of Deputies. The share of votes to the Five Star Movement (M5S) in the 2018 election is used as a counterfactual since the Prime Minister of Italy during the pandemic, Giuseppe Conte, was vastly supported from the start of its appointment in 2018 by the M5S. We argue that Prime Minister Giuseppe Conte’s leading political party is the Five Star Movement (M5S), and he ended up being the “face” of all the significant decisions that dealt with the pandemic. Thus we use the provincial vote share of M5S in the 2018 election as a proxy for the political support for the current legislation and its strategy to contain the virus throughout the epidemic. The vote share won by extreme right-wing party Lega, by contrast, is used as a measure of political misalignment and protest orientation of a particular province because of its deliberate opposition to Conte’s Prime Ministry in August 2019.^15^ Additionally, we use the share of null-votes to examine the effect of a protest vote and political disbelief on social distancing. As additional controls, we also add the provincial vote shares to Forza Italia and PD, the two second largest right-wing and left-wing parties. Such inclusion allows us to isolate the effect of more moderate right and left-wing political orientation on social distancing.

**Fig. 1:**
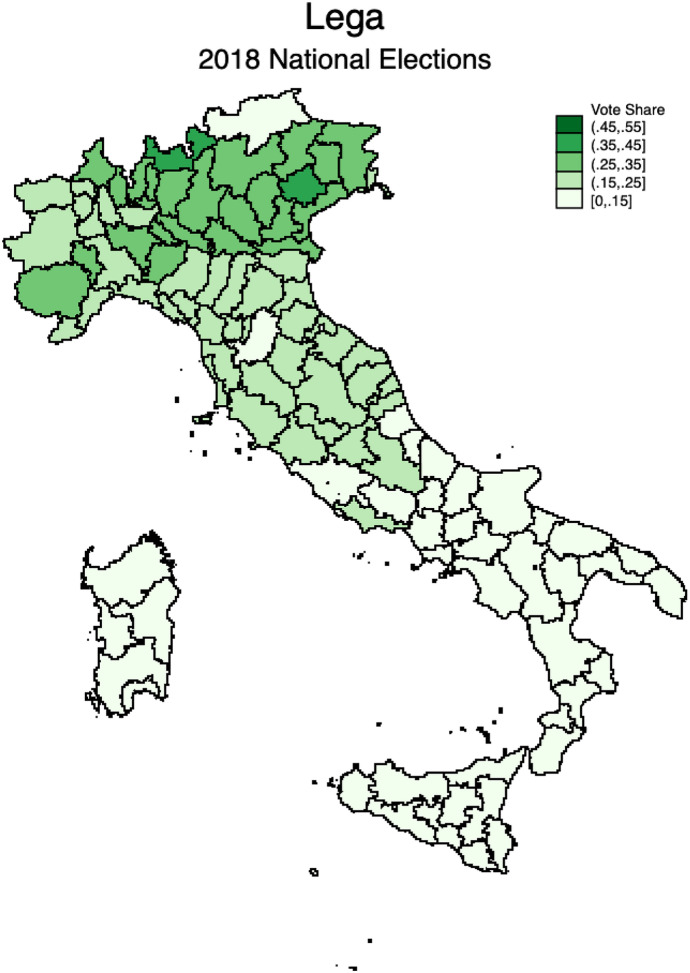
Provincial vote share (pps) for Lega in the 2018 National Election

**Fig. 2:**
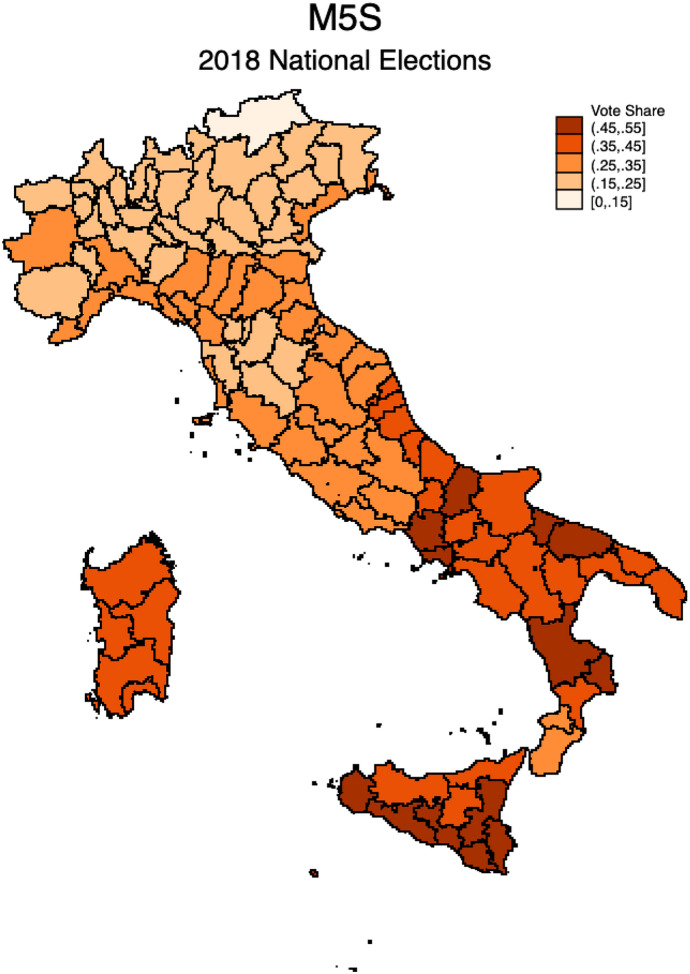
Provincial vote share (pps) for M5S in the 2018 National Election

**Fig. 3:**
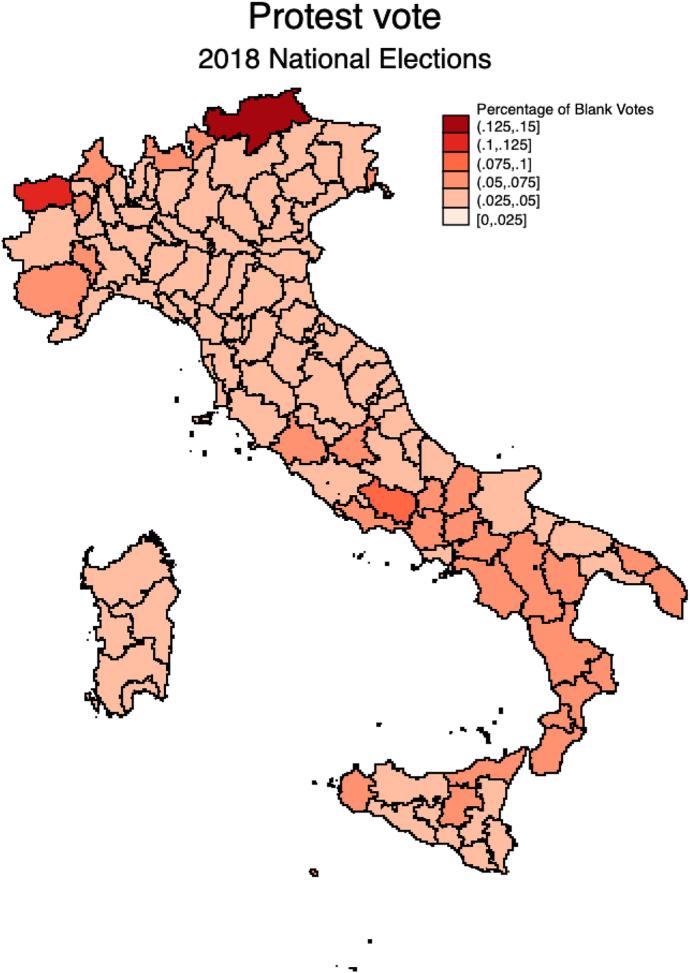
Provincial protest vote share (pps) in the 2018 National Election

Figures 1, 2, and 3 in Appendix show the vote share of Lega, M5S, and protest vote in the 2018 election by Italian provinces. It can be noticed that M5S gained a higher vote share overall, averaging around 30%, with over 40% shares in several Center/South regions; Lega instead gained a far less widespread vote share, averaging just around 15%, and more concentrated in the Northern regions; lastly, protest vote share was reasonably uniform, averaging around 5% with a few outliers.

Other controls To account for confounders related to the severity of the pandemic, we control for the number of new cases and the number of deaths (at the regional level) available by the official data provider for Italy, the Italian Civil Protection Department^16^.

In addition to this, we include several provincial/regional controls. We include data on the number of hospital beds (regular and intensive care) from the Italian Health Ministry, which might affect the perceived risk and cost of contracting COVID-19 and the individual incentive to social distancing. We also control for a rich set of geographic, socio-demographic, and economic factors that potentially correlate with both mobility and political orientation: area ($km^2$) and altitude of the province, population density, income per capita, added value from workers per capita, a dummy variable for the presence of an airport to capture an additional vulnerability to the pandemic. Since both political orientation (and voting pattern), as well as social proximity, are highly correlated with the size of cities and not just the size of provinces, we add a control for the distribution of the population by city size within provinces by including the percentage of the population that lives in cities with over 50000 people.^17^ This will allow us to control for the within-province heterogeneity that can drive both voting patterns and compliance with social distancing rules. We also add several demographic variables, at the provincial level, given that extensive evidence on previous pandemics show that demographic characteristics are determinants of the type and magnitude of protective behaviors (Bish and Michie 2010). To control for all of those characteristics, we add controls for the share of (provincial) female and immigrant populations. Including the share of the immigrant population also allows us to control, to some extent, the effect of globalization on electoral results, which has proven to have affected both far-right-wing voting (Caselli et al. 2019) and the Five-Star Movement (Caselli et al. 2020). We also add local labor market controls as employment rate and bachelor degree share, both taken from the 2011 population census. These variables not only allow us to control for any provincial heterogeneity in labor market outcomes, but they are also essential to account for the fact that local features play an important role when traveling bans were lifted.^18^ Lastly, to isolate the effect of civic capital, which might be correlated with voting shares in a particular province, we follow previous works and incorporate a measure of newspaper readership^19^ (Durante et al. 2020; Putnam et al. 2000; Guiso et al. 2004). This variable indicates the degree of public awareness of residents, which might affect civic engagement via information acquisition to improve collective decision-making quality.

## Econometric model

We examine the relationship between political (mis-)belief and social distancing using the following generalized difference-in-differences estimation:

1 $$\begin{aligned} p_{ijt} =\beta _{t} (Phase_t \times z_{ij})+\gamma _t (Phase_t \times x_{ijt})+\gamma _i+\gamma _t+\varepsilon _{it} \end{aligned}$$

where $p_{ijt}$ is the average daily proximity index for day *t* in province *i* in region *j*; $z_{ij}$ represents the proxy for the political belief in province *i* in region *j* and can be either the 2018 provincial-level vote share that went to Lega or M5S, or that was left blank, which in all regressions is interacted with a vector of dummies, $Phase_t$, representing the various phases of the pandemic which are: (1) lockdown (March 8–May 4); (2) phase 2 (May 4–June 16) and (3) phase 3 (June 16–June 26).^20^ We z-score $z_{ij}$ vote shares to have a mean of zero and a standard deviation of one. The $\beta$ coefficient will capture the marginal response to the different phases of the pandemic order based on how much a province leans towards different political parties. $x_{ijt} \times Phase_t$ are the interactions between the dummies for the different phases and all the economic, geographic, and demographic provincial controls described above. Including these terms allows to control for any differential changes in social distancing due to other characteristics of the province which are potentially correlated with political belief. $\gamma _i$ and $\gamma _t$ are province and time fixed effects. The province dummies absorb any systematic difference in mobility levels across provinces due to time-invariant characteristics. The day fixed effects account for the common trend of mobility on all provinces in any given day, absorbing any nation-wide information about the evolution of the epidemic. We cluster standard errors at the province level.

While we have taken careful steps to mitigate confounding factors, there still exists some potential for endogeneity. We cannot rule out that the decision of implementing the different phases may be endogenous to other (previous) social distancing recommendations implemented sparsely and asymmetrically in the different Italian regions, depending on how much the pandemic was affecting them. However, we try to account for this possibility through our day fixed effects and controls for the number of cases and deaths which captures how seriously the pandemic hit a region. Our identifying assumption is that, after controlling for any time-invariant province characteristic, nation-wide daily changes in mobility, and intensity of the pandemic at the local level, a differential change in social distance in a particular area is unrelated to factors other than the ones we explicitly control for. Finally, we cannot rule out the possibility that there is still some room left for endogeneity due to the omission of some socio-demographical controls correlated with voting behaviors.^21^

## Results

Columns (1) and (3) of Table 1 report baseline results. In Table 1, columns (2) and (4), we examine the same relationship over various phases. In all specifications, we control for province and day fixed-effects, the number of recent COVID-19 cases and deaths, and a complete set of controls described earlier interacted with the binary indicators for the pandemic’s different phases.^22^

We first examine whether, at the beginning of the coronavirus epidemic, Salvini’s confused speeches on the management of the pandemic influenced his voters’ behaviors. The Lega’s spokesman first dismissed the pandemic, demanding a softening or even entire cancelation of the lockdown (late February), supporting and disseminating the international fake news circulating on the social networks according to which the virus was artificially created in a Chinese lab in Wuhan. He then admitted to underestimating the situation and agreed on the lockdown (March). Not long afterward, he called for a faster re-opening (April), downplaying once again the seriousness of coronavirus and criticizing government’s pandemic containment measures.^23^ Even amid a health emergency, Salvini’s noncooperation attitude was immediately cticized by both the scientific and general public for gerrymandering.^24^ Therefore, it is reasonable to assume that right-wing extremists exhibit a lower sense of civic duties and have lower other-regarding preferences, affecting their compliance to lockdown. Also, we look at the effect of M5S vote share on social distancing to study how political representativeness drives mobility decisions. Since June 2018 and throughout the pandemic, Italy’s Prime Minister has been Giuseppe Conte, who, even if initially appointed by Lega and M5S, was supported during the first phase of the pandemic by a broader political audience (i.e., M5S and Democratic Party)

Consistently with such evidence, we find that during the sample period, the extra drop in social proximity was lower for provinces with a higher vote share towards Lega (Table 1, column (1)) than for provinces with a higher vote share towards M5S (Table 1, column (1)). Specifically, a one standard deviation increase in the vote share to Lega is associated with a -0.0037 extra decrease in the average degree of provincial proximity with respect to a -0.0048 decrease due to a one standard deviation increase in the vote share for M5S (Table 1 column (1)). This suggests that political support for M5S can significantly decrease mobility. Since we include both vote shares for M5S and Lega in the same model, we performed a Wald test to see whether their coefficients, as represented in Table 1 columns (1) and (2), differ. In both tests, the p-value was below 0.05,^25^ allowing us to reject the null hypothesis that the two coefficients are equal. Including the time indicators for phases two and three of the pandemic confirms the initial result (Table 1 column (2)), also showing that average proximity was more rapidly re-established in provinces with a higher vote share towards M5S. This can be reconciled with the fact that the provincial distribution of M5S vote share is concentrated among the Centre-South part of Italy (Figure A2), where a “new normal” after the pandemic was more rapidly reached, and the lower death toll that the epidemic registered.

**Table 1:** Social proximity for COVID-19, political affiliation

	(1)	(2)	(3)	(4)
Lockdown × Lega vote share	$$-$$0.00368$$^{***}$$	$$-$$0.00217$$^{***}$$		
	(0.000581)	(0.000708)		
Lockdown × M5S vote share	$$-$$0.00476$$^{***}$$	$$-$$0.00300$$^{***}$$		
	(0.000884)	(0.00105)		
Phase 2 × Lega vote share		0.00197$$^{***}$$		
		(0.000666)		
Phase 3 × Lega vote share		0.00556$$^{***}$$		
		(0.000975)		
Phase 2 × M5S vote share		0.00222$$^{**}$$		
		(0.000958)		
Phase 3 × M5S vote share		0.00670$$^{***}$$		
		(0.00138)		
Lockdown × null-vote share			0.00216$$^{***}$$	0.00146$$^{***}$$
			(0.000299)	(0.000358)
Phase 2 × null-vote share				$$-$$0.000978$$^{***}$$
				(0.000328)
Phase 3 × null-vote share				$$-$$0.00237$$^{***}$$
				(0.000479)
$R^{2}$	0.795	0.796	0.795	0.795
Time FE	Yes	Yes	Yes	Yes
Province FE	Yes	Yes	Yes	Yes
COVID-19 death and cases	Yes	Yes	Yes	Yes
Other controls × phase	Yes	Yes	Yes	Yes
N	13268	13268	13268	13268

Standard errors in parentheses

Standard errors are adjusted for clustering at province level

*$$p<.10$$, **$$p<.05$$, ***$$p<.01$$

Another factor that could have influenced individuals’ respect for social distancing orders is anti-establishment and protesting attitudes. Voting for anti-establishment parties is associated with a lower prosocial attitude, which could, in turn, undermine individual civic responsibility and predisposition to cooperate. Table 1 shows that a one standard deviation increase in provincial protest vote share is associated with an extra increase in the average network proximity by 0.0022 (Table 1, column (3)), with respect to the average over the same period.This effect then decreases during the two subsequent phases. This result contrasts an early finding and confirms the tendency that protest voters have for anti-establishment attitudes, even during a pandemic.

Overall, we find that political extremism and disillusion have the potential to undermine civic sense during the pandemic, making individuals less cooperative in decreasing mobility. Given such low compliance, voluntary social distancing driven by other-regarding considerations may not be sufficient to optimally contain the pandemic in the long-run, especially in Italian regions where political orientations are more extreme than the average. On the contrary, being more oriented towards the current legislation can enhance willingness to accept restrictions on one’s freedom to contain the virus spread.

## Conclusions

In this paper, we looked at the effect of political orientation (and disillusion) on compliance with social distancing and lockdown orders during the COVID-19 pandemic in Italy. Our results suggest that individuals with an extreme right-wing or protesting political orientation exhibit a lower civic-sense and lack of compliance with social distancing. These individuals might limit the effectiveness of public policy regarding mandatory stay-at-home orders and thwart the government’s attempt to manage the externalities associated with the spread of the virus. This, in turn, suggests that they might be less likely to stick, for the benefit of others? health and their own, with voluntary social distancing, creating negative externalities for the future non-emergency containment of the virus. Although voluntary social distancing driven by other-regarding considerations may not be enough to contain the pandemic efficiently, it may allow for social distancing policies that are less taxing on the economy and more sustainable for more extended periods.

Our results also indicate that bipartisan support is crucial for nationwide social distancing interventions and reinforces citizens’ beliefs on preventive strategies to manage the pandemic. As such, news and communication biases about the seriousness of the COVID-19 should be avoided for both political and ideological purposes. No health issues and no news of COVID-19 should be politicized^26^ and used for ideological interests. There should be bipartisan support on serious health matters and targeted campaigns to encourage public health communication’s visibility and reliability. Besides, there should be higher coordinated efforts involving various institutions, media, and digital platform companies to popularize important science-based messages.

Finally, our results suggest that political and civic tendencies can either help or harm the pandemic’s containment. Even if our findings refer mainly to the peak of the emergencies when extreme actions were needed to stop contagion, the behavioral responses we depict are likely to play an even more significant role as countries begin designing strategies to co-exist with the virus. In the long-run, when restrictions ease, social distancing will be achieved via individual social responsibility. More cooperative regions with less populist political orientation could manage the risk of recent outbreaks more efficiently without resorting to stringent economic lockdowns in such a phase.

## Appendix

See Table 2 and Figs. 1, 2, 3.

**Table 2:** Summary statistics

	Mean	SD	Min	Max
Average proximity index (daily)	0.03	0.0	0.0	0.2
Crowding index average dwelling	2.57	0.2	2.2	3.4
Population (log)	12.94	0.7	11.3	15.3
Female population (log)	12.27	0.7	10.7	14.6
Active population (log)	11.98	0.7	10.4	14.3
Graduated population (log)	10.60	0.8	9.2	13.4
Immigrant share (Province)	0.08	0.0	0.0	0.2
Total hospital beds	13108.10	9323.6	451.0	34938.0
Hospital beds in IC	362.11	233.1	12.0	859.0
Newspaper	0.04	0.0	0.0	0.1
Airport	0.29	0.5	0.0	1.0
Area (Km sq.)	2823.02	1677.2	212.5	8277.8
Avg altitude	402.99	299.6	4.7	1754.7
Population density	263.58	367.5	38.9	2591.3
Income (per capita)	35474.26	3776.2	28650.1	42315.0
Added value (per capita)	25787.33	6750.5	15311.5	42116.4
COVID-19 cases (Province)	1423.99	2663.4	0.0	24300.0
COVID-19 cases (Region)	12703.41	21404.1	0.0	93587.0
COVID--19 deaths (Region)	1864.96	3858.2	0.0	16624.0
Lega vote share (2018 election)	0.17	0.1	0.0	0.4
M5S vote share (2018 election)	0.32	0.1	0.1	0.5
Protest vote share (2018 election)	0.05	0.0	0.0	0.1

## Footnotes

1 Such that: stay-at-home orders, lock-down restrictions, social distancing measures, and travelling bans.

2 As Cairney and Wellstead (2021) suggest the chain of trust can be summarized as follows: “People need to trust experts to help them understand and respond to the problem, politician to coordinate policy instruments and make choices about levels of coercion, and citizens as they cooperate to minimize infection”.

3 Analysing data from a recent survey, the authors also find sizeable differences in beliefs about personal risk and future evolution of the health and economic crisis.

4 The Chinese authorities had already suspended those from Wuhan.

5 With a decree of 18th March 2020, the President of the Council of Ministers appointed Domenico Arcuri as Extraordinary Commissioner to implement and coordinate the necessary measures for the containment and contrast of the COVID-19 epidemiological emergency.

6 The DCPM February 25, 2020, the DCPM March 1, 2020, the DCPM March 4, 2020, the DCPM March 8, 2020.

7 An additional measure adopted on March 22, jointly signed by the Minister of Health and the Minister of the Interior, bans any movement with both public or private means of transport towards any municipality.

8 By the end of May, five various forms of self-certification were developed and published on the Ministry of the Interior website.

9 The Prime Minister’s Office allows the reopening of stores for babies and children, bookstores, and stationery stores from April 14.

10 Pepe et al. (2020) analyze a de-identified, large-scale dataset from a location intelligence and measurement platform, Cuebiq Inc Data for Good program.^11^ Data is collected from anonymized users who have opted-in to provide access to their location data anonymously, through a GDPR-compliant framework. Location is collected anonymously from opted-in users through a Software Development Kit (SDK) included in partner smartphone applications. At the device level, iOS and Android operating systems combine various location data sources (e.g. GPS, wifi, beacons, network) and provide geographical coordinates with a given level of accuracy. Location accuracy is determined by the device and is variable, but it can be as accurate as 10 meters. Temporal sampling of de-identified users’ location is also variable and dependent on app/OS characteristics and on user behavioral patterns, but overall, it has a high frequency.

12 We decided to use the last index for two main reasons: (1), the daily fraction of users moving between Italian provinces did not suit our research interest since our main purpose is to measure the effect of political orientation on distancing and not on mobility between provinces; (2) the median and IQR of users’ radius of gyration in a province, is collected weekly (and not daily) thus limiting the variability of our analysis.

13 Multiple links are counted only once every hour.

14 Provinces with a smaller users sample are characterized by lower values of *k*

15 Given his close political orientation to M5S rather than with Lega, in August 2019 Conte’s Prime Ministry suffered a motion of no confidence by Salvini, after growing tensions within the majority. This result in a new round of consultation and a new democratic majority emerged, between M5S and the Democratic Party, which announced its favourable position on keeping Giuseppe Conte at the head of the new executive.

16 See https://github.com/pcm-dpc/COVID-19

17 Since electoral systems are granted based on a threshold of 15,000 inhabitants per city, in a different specification, we also tried 15,000 as a threshold for our percentage and the results remain the same.

18 Caselli et al. (2020) show that catching up with pre-Covid-19 patterns has been stronger in those areas where the labour force is relatively less exposed to the risk of contagion and less likely to work from home.

19 In particular, we use the average diffusion of paid daily newspapers by region weighted by the total adult population, available from ADS in 2018.

20 The pre-outbreak period was excluded from the analysis.

21 For example, Caselli et al. (2019, 2020) show that the electoral dynamics in Italy over the 1994-2008 period was affected by two other globalizing factors on top of immigrations flows (which we account for): globalization factors: the intensity of import competition from China and diffusion of robots.

22 We did not report the results from the estimation with the sole provincial and day fixed effect because they are qualitatively equal to the one reported in Table 1, but they are available upon request.

23 See https://www.repubblica.it/politica/2020/04/16/news/salvini_e_i_cambi_di_rotta_sulle_aperture_attivita_-254162048/ and http://library.fes.de/pdf-files/bueros/rom/16948.pdf

24 Lega’s spokesman attitude is perfectly aligned with the populism tendency to lean to a post-truth politics, which uses social media as a mouthpiece for fake news and alternative facts. This media use has the main intention of inciting fear and hatred of “the other” to justify discriminatory health policies for marginalised groups (Speed and Mannion 2017).

25 The actual results were 0.0484 for the model in Table 1 column (1) and 0.01885 for the model in column (2).

26 See for example Abbas (2020), providing supporting evidence that the COVID-19 was widely used for political and ideological purposes. For the specific case of Italy Lovari (2020) show how Italy suffered from an information crisis during the pandemic due to the politicization of information.
